# Increased Transfection of the Easily Oxidizable GC-Rich DNA Fragments into the MCF7 Breast Cancer Cell

**DOI:** 10.1155/2019/2348165

**Published:** 2019-02-05

**Authors:** Svetlana V. Kostyuk, Nadezhda N. Mordkovich, Natalya A. Okorokova, Vladimir P. Veiko, Elena M. Malinovskaya, Elizaveta S. Ershova, Marina S. Konkova, Ekaterina A. Savinova, Maria A. Borzikova, Tatiana A. Muzaffarova, Lev N. Porokhovnik, Nataly N. Veiko, Serguey I. Kutsev

**Affiliations:** ^1^Research Centre for Medical Genetics (RCMG), Moscow 115478, Russia; ^2^Bach Institute of Biochemistry, Biotechnology Research Center, Russian Academy of Sciences, Moscow 119071, Russia; ^3^N. I. Pirogov Russian National Research Medical University, Moscow 117997, Russia; ^4^I.M. Sechenov First Moscow State Medical University (Sechenov University), Moscow 119991, Russia

## Abstract

**Objective:**

Easily oxidizable GC-rich DNA (GC-DNA) fragments accumulate in the cell-free DNA (cfDNA) of patients with various diseases. The human oxidized DNA penetrates the MCF7 breast cancer cells and significantly changes their physiology. It can be assumed that readily oxidizable GC-DNA fragments can penetrate the cancer cells and be expressed.

**Methods:**

MCF7 cells were cultured in the presence of two types of GC-DNA probes: (1) vectors pBR322 and pEGFP and (2) plasmids carrying inserted human rDNA (pBR322-rDNA and pEGFP-rDNA). pEGFP and pEGFP-rDNA contained a CMV promoter and a fluorescent protein gene *EGFP.* ROS generation rate, accumulation of the DNA probes in MCF7, 8-oxodG content, expression of *EGFP* and *NOX4*, and localization of EGFP, NOX4, and 8-oxodG in MCF7 were explored. The applied methods were qPCR, fluorescent microscopy (FM), immunoassay, and flow cytometry (FCA).

**Results:**

When GC-DNA is added to the cell culture medium, it interacts with the cell surface. At the site of GC-DNA contact with the cell, NOX4 is expressed, and ROS level increases. The ROS oxidize the GC-DNA. When using the plasmids pEGFP and pEGFP-rDNA, an increase in the amount of the DNA *EGFP*, RNA *EGFP*, and EGFP proteins was detected in the cells. These facts suggest that GC-DNA penetrates the cells and the *EGFP* gene is expressed. Insertions of the rDNA significantly increase the GC-DNA oxidation degree as well as the rate of plasmid transfection into the cells and the *EGFP* expression level. In the nucleus, the oxidized GC-rDNA fragments, but not the vectors, are localized within the nucleolus.

**Conclusions:**

GC-rich cfDNA fragments that are prone to oxidation can easily penetrate the cancer cells and be expressed. The cfDNA should become a target for the antitumor therapy.

## 1. Introduction

In the 1940s, it was discovered that mammalian DNA not only is contained in the cell nuclei but could be also found in the serum of peripheral blood [1]. The human cell-free DNA (cfDNA) is known to be enriched with GC-pairs. Mean GC-pair content in cfDNA of healthy controls is 53.7% [2], whereas gDNA contains 42% of GC-pairs [3]. In pathology and under the action of harmful environmental factors, cfDNA becomes increasingly enriched with GC-rich motifs (GC-DNA) [4]. A hallmark of accumulation of GC-DNA as part of cfDNA can be two highly repetitive sequences, which are present in hundreds of copies in the human genome: mitochondrial DNA [5, 6] and ribosomal genes (rDNA) [7]. The rDNA is easier to use, because its abundance in the genome is constant and does not depend on the current state of the cell.

A several fold increase in rDNA content within cfDNA is observed in chronic pathologies followed by exaggerated cell death (ischemic heart disease, chronic arterial hypertension, and rheumatic arthritis [7–9]), as well as in case of a chronic exposure to ionizing radiation or smoking [10, 11]. In some cases, the content of rDNA fraction within cfDNA can increase by more than an order of magnitude.

As a result of the change in CG-composition of cfDNA observed in autoimmune and cardiovascular pathologies, the cfDNA becomes biologically active. Both models GC-DNA and cfDNA from the patients induce changes in the functional activity of human endothelial cells [12], rat cardiomyocytes [13], neurons [14], human stem cells [15], and lymphocytes [16]. The first and major sign of the GC-DNA impact is elevated ROS production [15].

In spite of intensive studies of cfDNA in oncological diseases [17], whether GC-DNA fragments possess biological activity in respect of cancer cells remains elusive. We showed previously that exposure to the oxidized human gDNA enhances both genome instability and survival in MCF7 cancer cells [18]. Nonoxidized human gDNA did not possess such properties. Since human GC-DNA contains a high number of most easily oxidizable dGn (*n* > 2) motifs [15], one can expect that these oxidized DNA fragments exhibit activity with regard to cancer cells.

The biological activity of oxidized human gDNA is manifested as a consequence of its more effective penetration into the cells [18]. GC-DNA can be also expected to penetrate easily the cells owing to its higher oxidation degree. Alongside with that, promotors of approximately 40% of human genes are known to include CpG islets (about 1.5 kbp long), which are identical to rDNA with respect to their GC-composition and could accumulate within cfDNA. The accumulation of a fraction of the genes with GC-rich promotors within cfDNA can result in the expression of these genes in the cells. In addition, DNA fragments, when penetrating the cells, can bind and exhaust the pool of factors that regulate the expression of some specific genes. As a result, the gene expression patterns can change.

Thus, in this study, we intended to obtain answers for the following questions: (1) Does the GC-DNA, containing rDNA, have an ability to penetrate MCF7 cancer cells? (2) Can the genes contained in the extracellular GC-DNA be expressed inside MCF7 cells? (3) Can the extracellular GC-DNA containing the genes modulate the expression of the same genes in the nucleus?

## 2. Methods

### 2.1. Cell Culture

ER/PR-positive MCF7 breast cancer cells were purchased at ATCC, Manassas, USA (Cat: HTB-22). MCF7 cells were cultured in DMEM medium supplemented with 10% (*v*/*v*) fetal calf serum, 2 mM L-glutamine, 100 units/mL penicillin, and 100 *μ*g/mL of streptomycin. Cells were grown in a humidified atmosphere with 5% CO_2_ in air at 37°C. Before the treatment with GC-DNA probes, cells were grown for 48 h in slide flasks.

### 2.2. Model GC-DNA

**Figure 1:**
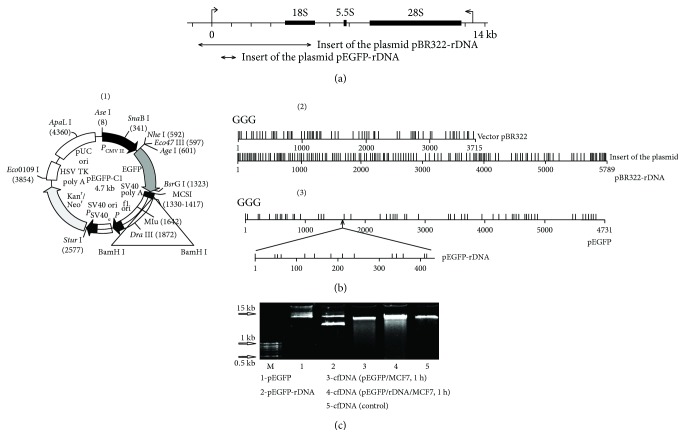
(a) Scheme of the human ribosomal repeat. Segments—the inserts of the model GC-DNA analyzed are shown. (b) (1)—plasmid pEGFP-C1 (pEGFP) (http://www.bdbiosciences.com, GenBank accession number U55763). The site BamH1 is marked. (2) and (3)—distribution of the easily oxidizable GGG-motifs within pBR322, pBR322-rDNA, pEGFP, and pEGFP-rDNA. (c) Determination of the plasmid fragmentation in the extracellular DNA after 1 h of MCF7 incubation with 50 ng/mL pEGFP or pEGFP-rDNA. The total DNA was isolated from the cell culture medium. Electrophoresis of DNA was carried out in a 2% agarose gel stained with ethidium bromide. Gel tracks 1 and 2—plasmid solutions in water were applied.

Plasmid pEGFP-C1 (pEGFP) that contains the EGFP gene (http://www.bdbiosciences.com, GenBank accession number U55763) was used as a vector (Figure 1(b)). The DNA fragment to be inserted was synthesized and consisted of 420 base pairs flanked with BamHI restriction sites and containing the rDNA. Cloned rDNA fragment covers positions from 601 to 1021 b of human rDNA (Figure 1(a)).

Plasmid pBR322 is the commercial product (Sigma-Aldrich). pBR322-rDNA (plasmid DNA) contains rDNA sequences cloned into the EcoRI site of pBR322 vector. Cloned rDNA fragment covers positions from −515 to 5321 of human rDNA (Figure 1(a)).

#### 2.2.1. Plasmid Clearance from Endotoxins

All the GC-DNA samples were subjected to the purification procedure removing lipopolysaccharides; this included sequential treatment with Triton X114 (Merck, Germany) followed by gel filtration on the HW 85 [19] or the use of an Endotoxin Extractor (Sileks, Russia). In order to prove that the observed response was caused exclusively by DNA, not by endotoxin residuals, additional experiments were set up. (1) A sample of plasmid DNA underwent complete hydrolysis down to nucleosides using DNA exonucleases and phosphatase. The resultant plasmid hydrolysates had no biological activity, which is intrinsic to an intact DNA.

(2) We analyzed the expression of TLR4 gene, which is always activated in the presence of the endotoxin. The samples of plasmid DNA induced no increase of TLR4 expression.

### 2.3. The cfDNA Samples Obtained from Blood Plasma

We used four blood plasma samples derived from healthy donors and four blood plasma samples from untreated breast cancer patients, who had applied for genetic tests in RCMG. The investigation was carried out in accordance with the latest version of the Declaration of Helsinki and approved by the Regional Ethics Committee of RCMG (Approval #5). All participants signed an informed written consent to participate after the nature of the procedures had been completely explained to them. CfDNA isolation from blood plasma and quantification of rDNA in the cfDNA was performed as described earlier [10]. The content of the transcribed region of rDNA was presented as pg rDNA/ng cfDNA.

### 2.4. Flow Cytometry

#### 2.4.1. EGFP

Nonfixed cells were analyzed at 488 nm. To quantify the background fluorescence, the control cells were analyzed.

#### 2.4.2. 8-oxodG and NOX4

Staining of the cells with antibodies was performed as previously described [18]. To quantify the background fluorescence, we stained a portion of the cells with secondary FITC- (PE) conjugated antibodies only.

Cells were analyzed at CyFlow Space (Partec, Germany).

### 2.5. Quantification of mRNA

Total mRNA was isolated using RNeasy Mini kits (Qiagen, Germany), treated with DNAse I, and reverse transcribed by a Reverse Transcriptase kit (Sileks, Russia). The expression profiles were obtained using qRT-PCR with SYBR Green PCR Master Mix (Applied Biosystems). The mRNA levels were analyzed using the StepOnePlus (Applied Biosystems); the technical error was approximately 2%. The following primers were used (Sintol, Russia):

1. EGFP (F TACGGCAAGCTGACCCTGAAG; R TGAAGCACTGCACGCCGTAGG)
2. NOX4 (F:TTGGGGCTAGGATTGTGTCTA; R:GAGTGTTCGGCACATGGGTA)
3. TBP (reference gene) (F: GCCCGAAACGCCGAATAT; R: CCGTGGTTCGTGGCTCTCT)

### 2.6. Quantification of pEGFP and pEGFP-rDNA in the Cells and Medium

#### 2.6.1. The Cells

After incubation medium removal by centrifugation at 460 *g*, cells were mixed with the solution (1 mL) containing 0.2% sodium lauryl sarcosylate, 0.002 M EDTA, and 75 *μ*g/mL RNAse A (Sigma-Aldrich, USA) and incubated for 45 min, then treated at 37°C with proteinase K (200 *μ*g/mL, Promega, USA) for 24 h. After two cycles of the purification with saturated phenolic solution, DNA fragments were precipitated by adding two volumes of ethanol in the presence of 2 M ammonium acetate. The precipitate was then washed with 75% ethanol twice, dried, and dissolved in water. The concentration of DNA was determined by measuring fluorescence intensity after DNA staining with the PicoGreen (Molecular Probes/Invitrogen, CA, USA). The contents of pEGFP and pEGFP-rDNA were obtained using qPCR with SYBR Green PCR Master Mix (Applied Biosystems). The following primers were used (Sintol, Russia):

1. *EGFP* (F: TACGGCAAGCTGACCCTGAAG; R: TGAAGCACTGCACGCCGTAGG)
2. Human B2M (reference gene, accession number M17987) (F: GCTGGGTAGCTCTAAACAATGTATTCA; R: CATGTACTAACAAATGTCTAAAATGG)

#### 2.6.2. Culture Medium

For the isolation of DNA from the cell culture medium, a procedure similar to that described above for the cells was used. DNA underwent electrophoresis in a 2% agarose gel stained with ethidium bromide.

### 2.7. 8-oxodG Levels in pEGFP and pEGFP-rDNA

#### 2.7.1. MCF7 1 h (Figure 2(c))

MCF7 were cultured in the presence of plasmids for one hour. The RNA fraction which contained fragments of plasmid DNA was isolated using YellowSolve (Sileks, Russia). RNA was digested (1 h, 37°, 75 *μ*g/mL RNAse A), and DNA was precipitated with 75% ethanol. The contents of pEGFP and pEGFP-rDNA were obtained using qPCR.

#### 2.7.2. *UV/H_2_O_2_* (Figure 2(c))

The method for DNA oxidation was specified previously [18]. Briefly, plasmids pEGFP and pEGFP-Gn (100 ng/*μ*L) were oxidized in 0.1% H_2_O_2_ solution with UV irradiation (*λ* > 312 nm) for 3 minutes at 25°C. Modified DNA was precipitated with two volumes of ethanol in the presence of 2 M ammonium acetate. The precipitate was washed twice with 75% ethanol, then dried, and dissolved in water. Resulting DNA concentrations were quantified by an analysis of the UV spectra.

The method for 8-oxodG quantitation was specified in details previously [20]. Briefly, the DNA samples were applied to a prepared filter (Optitran BA-S85, GE Healthcare). Three dots (10 ng/dot) were applied per each sample. Four standard samples of the oxidized genomic DNA (10 ng/dot) with a known content of 8-oxodG (determined by ESI-MS/MS using AB SCIEX 3200 Qtrap machine [18]) were applied onto the same filter, in order to plot a calibration curve for the dependence of the signal intensity on the number of 8-oxodG copies in a particular sample. The filter was heated at 80°С in vacuum for 1.5 h. 8-oxodG antibody conjugated with alkaline phosphatase was used. Then the filter was placed into a solution of substrates for alkaline phosphatase NBT and BCIP. Upon the completion of reaction, the filter was washed with water and dried in the darkness. The dried filter was scanned. For the quantitative analysis of the dots, special software was used (Images6, RCMG, Moscow). Signals from several dots for the same sample are averaged. The 8-oxodG content in a studied sample is calculated using the calibration curve equation. A relative standard error was 15% ± 5%.

### 2.8. Fluorescence Microscopy

#### 2.8.1. Immunocytochemistry

MCF7 cells were fixed in 3% formaldehyde (4°C) for 20 min, washed with PBS, and then permeabilized with 0.1% Triton X-100 in PBS for 15 min at room temperature, followed by blocking with 0.5% BSA in PBS for 1 h and incubated overnight at 4°C with the NOX4 and 8-oxodG antibody. After washing with 0.01% Triton X-100 in PBS, MCF7 cells were incubated for 2 h at room temperature with the FITC/PE goat anti-mouse IgG, washed with PBS, and then stained with DAPI.

#### 2.8.2. Intracelullar Localization of Labeled GC-DNA Fragments

Labeling of pBRR322 and pBR322-rDNA was performed by nick translation using a CGH Nick Translation Kit (Abbott Molecular) under the manufacturer's protocol. Labels pBR322^green^ and pBR322-rDNA^green^ or pBR322-rDNA^red^ were added to the cultivation media for 30 min. Cells were washed three times with PBS, fixed in 3% paraformaldehyde (4°C) for 10 min, washed with PBS, and stained with 2 *μ*g/mL DAPI.

#### 2.8.3. EGFP

Nonfixed cells were analyzed at 488 nm. To quantify the background fluorescence, the control cells were analyzed.

#### 2.8.4. Mitochondria

The cells were stained with 30 nM TMRM (tetramethylrhodamine methyl ester) (Molecular Probes) for 20 min at 37°C.

### 2.9. ROS Assay

The cells were analyzed using a total fluorescence assay in the 96-well plate format at *λ*ex = 488 nm and *λ*em = 528 nm (EnSpire equipment, Finland). The cultivation medium was replaced by 5 *μ*m H_2_DCFH-DA (Molecular Probes/Invitrogen, CA, USA) in PBS solution, and a relative fluorescence intensity increase was detected at 37°C. 16 (8 × 2) repeated measurements were provided for each GC-DNA concentration, and 24 for the control. The mean absolute intensities were divided by the average value of the intensity corresponding to *t* = 0, obtaining the values of *I*_0_. The graphs are presented in the coordinates *I* − time. The obtained data were approximated by linear dependence; the value of the tangent of the slope (index *k*_i_) together with the error of determination was calculated. *k*_0_ is reaction rate constant for DCF formation in control cells: Δ*k* = *k*_i_ − *k*_0_.

### 2.10. Statistics

All reported results for qPCR, PT-qPCR, immunoassay, and FCA were reproduced at least three times as independent biological replicates. The significance of the observed differences was analyzed using nonparametric Mann–Whitney *U* tests. The data were analyzed with StatPlus2007 Professional software (http://www.analystsoft.com/). All *p* values were considered statistically significant at *p* < 0.05. The software for “Imager 6” was designed by R. Veiko (RCMG, Moscow).

## 3. Results

### 3.1. Experimental Design

In order to study the eventual biological activity of GC-DNA towards MCF7 cancer cells, four model GC-DNA constructs were used (Figures 1(a) and 1(b)):

1. pBR322-rDNA: plasmid DNA (10,197 bp) contains rDNA sequences (5836 bp, 73% GC) cloned into the EcoRI site of pBR322 vector. The rDNA fragment clone covers the positions from −515 to 5321 of human rDNA (Figure 1(a))
2. pBR322: vector (4361 bp, 53% GC) served as a control for pBR322-rDNA
3. pEGFP-rDNA: plasmid DNA (5151 bp) contains GC-rich rDNA sequences (420 bp, 91.9% GC) cloned into the BamH1 site of pEGFP-C1 vector. Cloned rDNA fragment covers positions from 601 to 1021b of human rDNA (Figure 1(a))
4. pEGFP: plasmid pEGFP-C1 (53.4% GC) contains EGFP gene used as a control for pEGFP-rDNA

**Figure 2:**
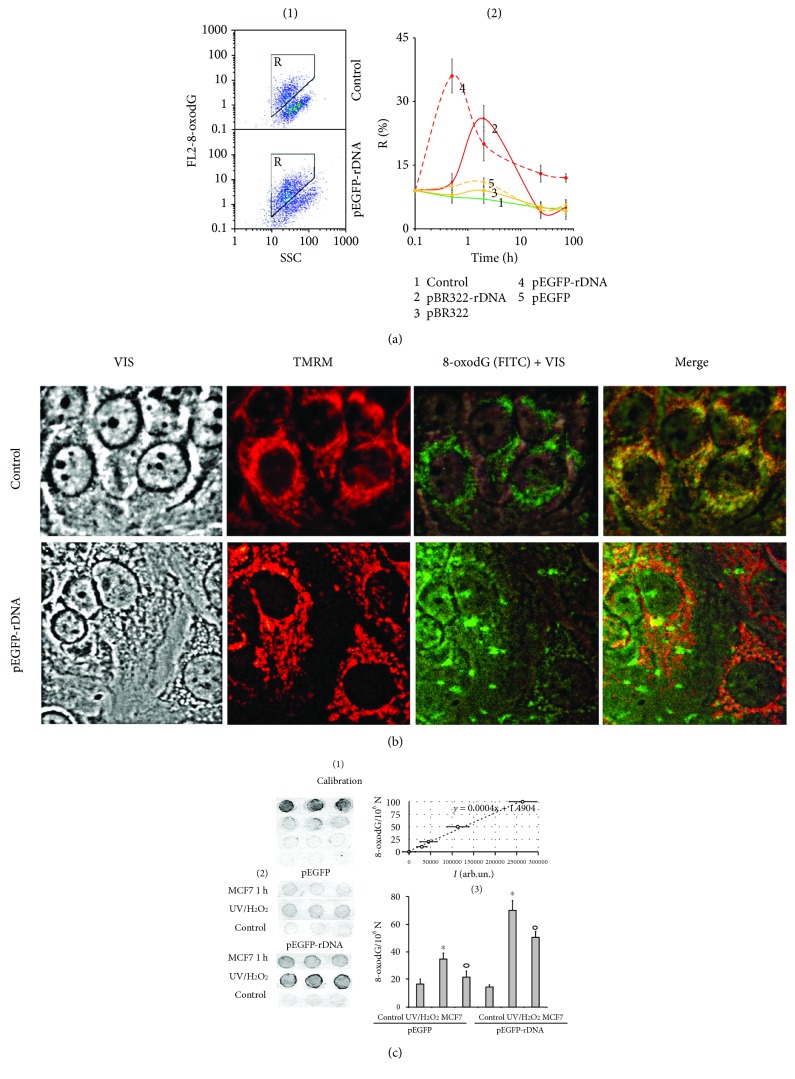
8-oxodG levels in MCF7. (a) FCA: (1)—cell plots: FL2 (8-oxodG-PE) versus SSC. R: gated area. (2)—relative proportions of 8-oxodG-positive cells in R gate (change with time). (b) FM-based evaluation of mitochondria and 8-oxodG (FITC) in the cells treated with pEGFP-rDNA for 1 h (×40). Unfixed cells were stained with MitoTracker TMRM (15 min, 37° C) and photographed. Next, cells were fixed with 3% paraformaldehyde, treated with 0.1% Triton X100, and 8-oxodG was detected using antibodies (FITC). Photographed in the same field. (c) 8-oxodG levels in pEGFP and pEGFP-rDNA. Immunoassay technique on nitrocellulose membranes using 8-oxodG antibodies conjugated with alkaline phosphatase was used. (1 and 2)—four standard samples of oxidized genomic DNA (10 ng/dot) with a known content of 8-oxodG (was determined by ESI-MS/MS using AB SCIEX 3200 Qtrap machine [18]) were applied in order to plot a calibration curve for the dependence of the signal intensity on the number of 8-oxodG bases. (3 and 4)—the samples of oxidized and nonoxidized (control) pEGFP and pEGFP-rDNA (10 ng/dot) were applied. MCF7 1 h—pEGFP or pEGFP-rDNA after 1 h of incubation with MCF7; UV/H_2_O_2_—plasmids were oxidized in 0.1% H_2_O_2_ solution with UV irradiation (*λ* > 312 nm) for 3 minutes at 25°C (Vilber Lourmat equipment, TCP-20.LM).

**Figure 3:**
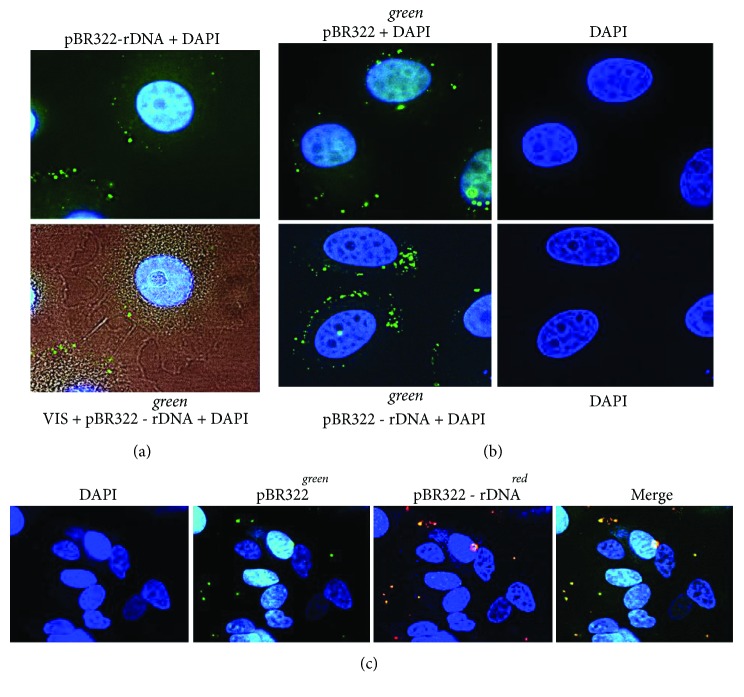
Staining of MCF7 cells with various types of labeled GC-DNA. (a) Top photo: pBR322-rDNA*^green^*, nuclei are stained with DAPI. Bottom photo: merged staining patterns of pBR322-rDNA*^green^*, DAPI and the image of the cell in visible light (×100). (b) pBR322*^green^* (top photo) and pBR322-rDNA*^green^* (bottom photo). (c) Merged staining patterns of pBR322*^green^* and pBR322-rDNA*^red^* (×40). Labeling of pBRR322 and pBR322-rDNA was performed by nick translation using a CGH Nick Translation Kit (Abbott Molecular). Labels pBR322*^green^* and pBR322-rDNA*^green^* or pBR322-rDNA*^red^* were added to the cultivation media (50 ng/mL) for 30 min. Cells were washed three times with PBS, fixed in 3% paraformaldehyde (4°C) for 10 min, washed with PBS, and stained with 2 *μ*g/mL DAPI.

All the plasmids are enriched with GC-pairs compared to human gDNA (42% GC). Plasmid sequencing showed multiple GGG motifs, Figure 1(b), (2) and (3). The dG bases included in such motifs are known to have the lowest oxidation potentials among all the bases in the DNA molecule [21].

In addition to plasmids, cfDNA samples isolated from the blood plasma of healthy donors (*N* = 4) and the blood plasma of untreated breast cancer patients (*N* = 4) were also used. In the cfDNA samples, rDNA content was determined. The cfDNA samples derived from the cancer patients contained more rDNA, than the samples from healthy donors (1.3–2.1 pg/ng cfDNA vs 3.1–4.2 pg/ng cfDNA).

The MCF7 culture medium contained 320 ± 40 ng/mL cfDNA 48 h after the start of culture growth. The rDNA content in gDNA and cfDNA was, respectively, 1.47 pg/ng gDNA [22] and 2.5 pg/ng cfDNA. GC-DNA was added to the MCF7 culture medium in a concentration of 50 ng/mL. So the total cfDNA content slightly increased (by a factor of 1.15, up to 370 ± 40 ng/mL), while the GC-rDNA content substantially elevated (approximately 20-fold compared to the cfDNA content in the control cells). The cells were cultured with GC-DNA for 0.5 to 72 h.

A question whether plasmid DNA requires linearization before adding to the culture medium was considered specially. Our first tests failed to show any difference between the rates of accumulation of intact GC-DNA and their linearized forms in the cells. We examined the conformation of plasmids, which had remained in the medium after one-hour cell incubation, using electrophoresis in a 2% agarose gel (Figure 1(c), data for pEGFP and pEGFP-rDNA are presented). The initial plasmid pool contained various circular forms (tracks 1 and 2, Figure 1(c)). However, following incubation with the cells, almost only one form remained (tracks 3 and 4), which was partially fragmented. Thus, when the cells were present in the medium, the supercoiled circular plasmid DNA is rapidly hydrolyzed down to linear molecules of different length that roughly corresponds to the length of fragments of cell's own cfDNA accruing in the culture medium (track 5). On the basis of these data, we added nonlinearized plasmids to the culture medium during the major experiments.

### 3.2. Interaction of GC-DNA with the Cells

The probes pBR322-rDNA^green^, pBR322-rDNA^red^, and pBR322^green^ were labeled with SpectrumRed and SpectrumGreen [18]. After 30 minutes of incubation with MCF7, BR322-rDNA^green^ and pBR322^green^ demonstrated approximately the same MCF7 binding pattern: the signals were situated as separate grains over the cytoplasm periphery (Figures 3(a) and 3(b)) and could be observed approximately in half of the cells. More detailed simultaneous analysis showed that GC-DNA binding to the cell depended on the base sequence (Figure 3(c)). Not all signals observed in the cell after binding to pBR322-rDNA^red^ and pBR322^green^ probes coincide: signals from pBR322-rDNA^red^ probe are more numerous. As early as 3 h after, the fluorescence of DNA-probed in MCF7 considerably decreased and was not detected at all 24 h later.

Thus, firstly, rDNA fragments selectively interact with some cellular structures and, secondly, fluorescent label in GC-DNA probe quickly degrades. The latter can suggest an elevated level of ROS, which oxidize and/or quench the dye fluorescence.

### 3.3. GC-DNA Induces a Transient Oxidative Stress in MCF7

**Figure 4:**
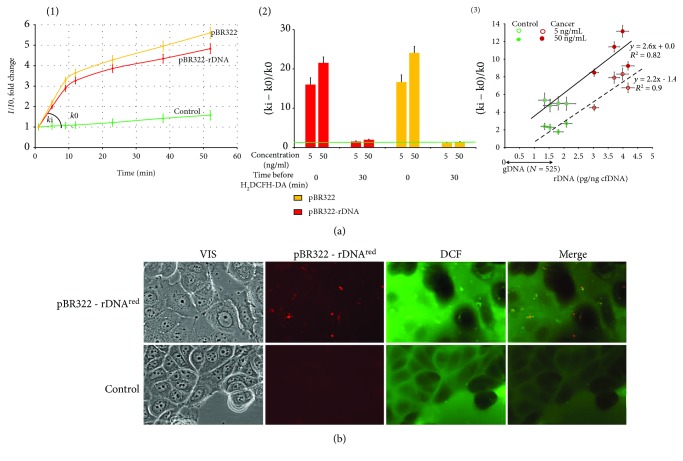
The exposure of MCF7 cells to GC-DNA leads to an increase in ROS production. (a) FL-plate reader. The cells were analyzed using total fluorescence assay in the 96-well plate format at *λ*_ex_ = 488 nm and *λ*_em_ = 528 nm (EnSpire equipment). (1)—an example of reaction rate constant determination for DCF formation. The cultivation medium was replaced with 5 *μ*m H_2_DCFH-DA in PBS solution, GC-DNA was immediately added to the solution (50 ng/mL), and a relative fluorescence intensity increase was detected at 37°C. *I* and *I*_0_—sample's signal at time *t* and immediately after H_2_DCFH-DA and GC-DNA addition, respectively. The line slope-reaction rate constant for DCF formation (*k*). ROS index Δ*k*/*k*_0_ = (*k*_i_ − *k*_0_)/*k*_0_. (2)—ROS index for pBR322-rDNA and pBR322. Time of cultivation with GC-DNA before adding H_2_DCFH-DA and plasmid concentration is shown in the figure. (3)—dependence of ROS index on the rDNA content in cfDNA samples. The cfDNA samples (5 or 50 ng/mL) were added after H_2_DCFH-DA addition. (b) FM-based evaluation of MCF7 cells sequentially treated with 5 *μ*m H_2_DCFH-DA and pBR322-rDNA*^red^* (50 ng/mL) and incubated for 30 min (×40). Top photo: red granules—pBR322-rDNA localization in the cells; green granules—synthesis of DCF. Some of the signals are the same, indicating DCF synthesis at the site of DNA contact with the cell. In the presence of pBR322-rDNA, it increases the overall intensity of green fluorescence, compared with the control (bottom photo).

To study the possible influence of GC-DNA on the intracellular levels of ROS, the ROS were measured using dichlorodihydrofluorescin diacetate (H_2_DCFH-DA) dye that rapidly penetrates cell membranes and gets trapped in the cytosol in its deacetylated form. In the cytosol, nonfluorescent DCFH serves as a sensitive intracellular marker for oxidative stress upon its oxidation to DCF by a variety of ROS [23].  Figure 4(a) shows the results of ROS assay in living cells using a plate reader.

When we added DNA samples after adding H_2_DCFH-DA to the medium, we observed a signal increase in the presence of GC-DNA compared to the control. The peak rate of DCF production was observed within the first 20 min after adding GC-DNA to the cells (Figure 4(a)). Later, the curve slope diminished several times. But if the cells were at first exposed to DNA samples during 30 min and then added H_2_DCFH-DA, the effect lacked (Figure 4(a), (2)).

Both cfDNA samples from controls and patients stimulated ROS synthesis in the cells. The cell response for the exposure to the cancer cfDNA samples was higher than for the exposure of the control cfDNA and comparable to the cell response for the exposure to the model GC-DNA. The ROS synthesis level positively correlated with the rDNA content in cfDNA (Figure 4(a), (3)).

Figure 4(b) displays the results of ROS assay in the living cells using fluorescence microscopy. H_2_DCFH-DA dye stains the control cells mainly along the surface of the cell membrane. GC-DNA initiates stained grains in the cytoplasm. The localization of green DCF grains mainly coincides with the localization of red grains of the tagged DNA-probe GC-DNA^red^ (Figure 4(b)). It seems that the interaction of GC-DNA with the cellular structures stimulates ROS production in the place of the contact.

One can conclude, firstly, that GC-DNA is an inducer of quick ROS synthesis in MCF7 cells. Secondly, in parallel with the active ROS production, a process is initiated in the cells, which is aimed at reducing the ROS level.

### 3.4. GC-DNA Evoked an Increase in NOX4 Expression

**Figure 5:**
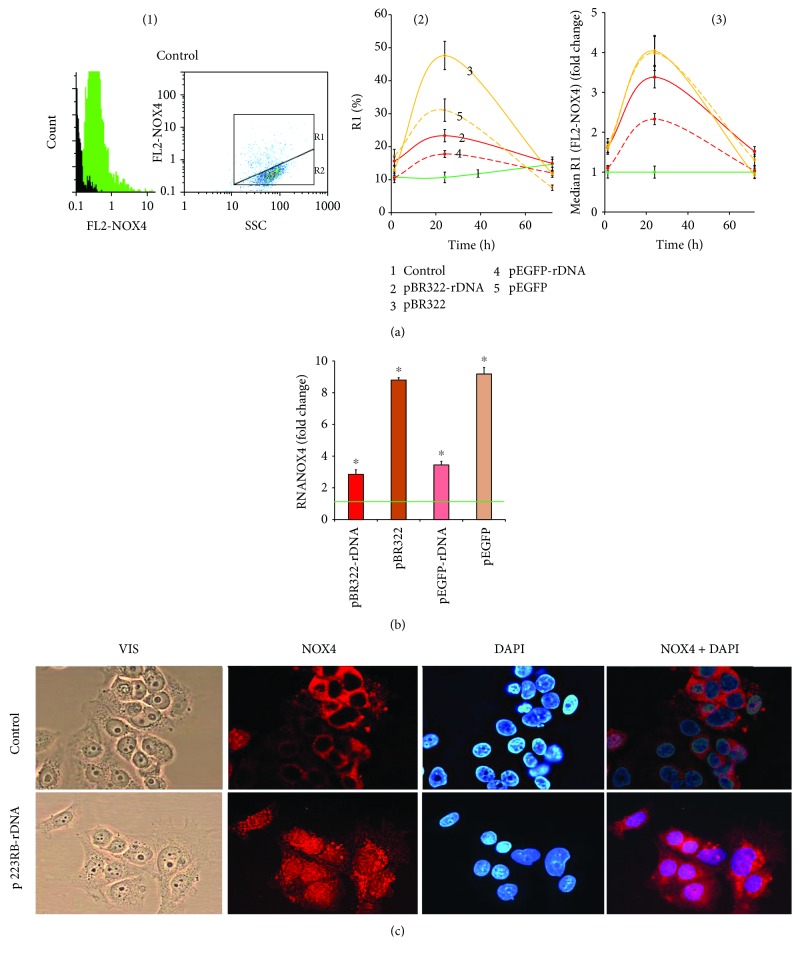
Expression of *NOX4* in MCF7. (a) FCA. (1)—the distribution of FL2-NOX4 fluorescence intensities; black: background fluorescence (PE-conjugated secondary antibodies). Cells plots: FL2 (NOX4-PE) versus SSC; R1 and R2: gated areas. (2)—relative fractions of NOX4-positive cells in R1 gate. (3)—median signal intensity of FL2-NOX4 in R1 gate. (b) RT-qPCR. The levels of NOX4-encoding RNAs in the cells exposed to GC-DNA for 24 h. The data are normalized by the content of RNA *NOX4* in the control. (c) FM-based evaluation of NOX4 (PE) in the cells treated with pEGFP-rDNA for 24 h (×40). ^∗^*p* < 0.05 against control group of cells (nonparametric *U* test).

The amount of NOX4 protein in the cells was evaluated using FCA (Figure 5(a)). MCF7 cell culture harbors two cell subpopulations: with high (gate R1, approximately 10% of the cells) and low (gate R2) contents of NOX4 protein (Figure 5(a), (1)). GC-DNA induced an increase of NOX4 amount. The effect was maximum in 24 h after the start of exposure and decreased by 72 hours down to the baseline. The pEGFP and pBR322 plasmids that carried no rDNA inserts evoked an increase of the rate of cells with high level of NOX4 expression by a factor of two and five, respectively. The plasmids that carried rDNA inserts stimulated the NOX4 protein synthesis in a less degree.

The level of RNA *NOX4* in 24 h after an exposure to GC-DNA is shown in Figure 5(b). In the presence of pEGFP-rDNA and pBR322-rDNA plasmids, the RNA *NOX4* amount increased by a factor of 2 to 4, whereas in the presence of pEGFP and pBR322, increased by a factor of 9 to 10.

In control cells, NOX4 is located on the cellular surface and in the cytoplasm. In the presence of GC-DNA, this protein is expressed on the cellular surface, in the cytoplasm, and in the nucleus (Figure 5(c)).

Thus, all the types of GC-DNA induced an increase in *NOX4* expression; however, the presence of rDNA inserts reduced this effect.

### 3.5. GC-DNA Induced an Increase in 8-oxodG Content in the Cells

The 8-oxodG content in the cells was determined using FCA (Figure 2(a)). pEGFP-rDNA and pBR322-rDNA increased the fraction of cells with high 8-oxodG content (gate R, Figure 2(a), (1)) by a factor of 2-3 (*p* < 0.05). The effect reached the peak in the first several hours and decreased in 24 h (Figure 2(a), (2)). An exposure to the plasmids pEGFP and pBR322 slightly influenced the 8-oxodG content in the cells (*p* > 0.05).

**Figure 6:**
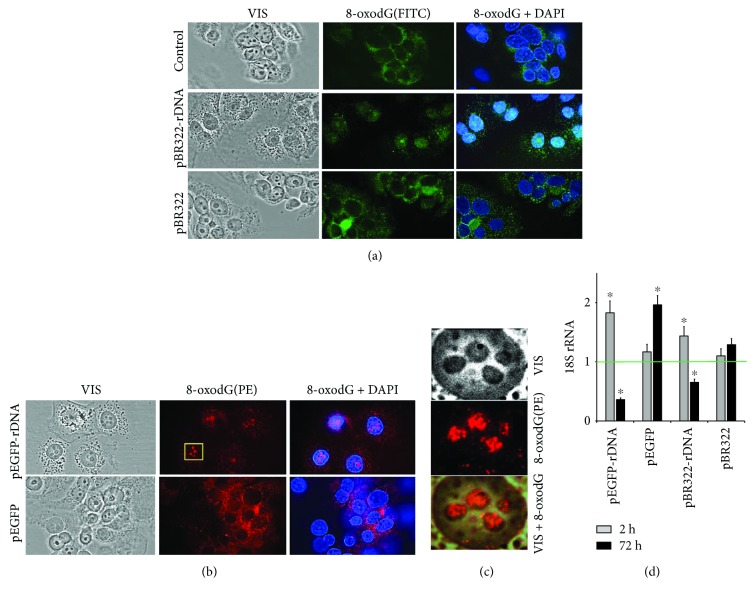
FM-based evaluation of 8-oxodG in the cells treated with pBR322 and pBR322-rDNA (a) and pEGFP and pEGFP-rDNA (b) for 24 h (×40). (c) For the analysis of the selected area of the photo, the image size was increased via computer processing. (d) RT-qPCR. The levels of 18S rRNA in the cells exposed to GC-DNA for 2 h and 24 h. The data are normalized by the content of 18Sr RNA in the control.

The elevation of intracellular 8-oxodG signal after an exposure to pEGFP-rDNA and pBR322-rDNA within the first hours can be associated with an increased oxidation level of the cellular DNA (mitochondrial and nuclear) and/or with an increased oxidation level of the plasmid itself followed by interaction with the cells. In order to elucidate this question, we determined the localization of 8-oxodG signals in the cells using fluorescence microscopy (Figures 2(b) and 6). In the control cells, 8-oxodG (FITC) was detected in the mitochondria (MitoTracker TMRM). Within the first 30 minutes after the beginning of exposure to GC-DNA, additional compact single signals were detected in the cellular cytoplasm. Their localization only partially coincided with the mitochondria. These signals were more abundant in case of exposure to pEGFP-rDNA and pBR322-rDNA. 24 h later, these signals were not detected. Signals from 8-oxodG detected in the mitochondria insignificantly differed from the control sample by intensity.

Thus, the increase of 8-oxodG content in the cells (Figure 2(a)) is induced by an elevated oxidation level of GC-DNA itself. It should be noted that the cell treatment before FCA (washing with a solution containing trypsin and EDTA) led to detaching the superficially bound cfDNA and complexes of cfDNA with proteins from the cellular surface [24, 25]. Using FCA, it was possible to detect only those fragments of oxidized GC-DNA, which had already penetrated the cytoplasm through the cell membrane.

In order to find out finally the cause of the difference in 8-oxodG content between the cells exposed to vectors or to pEGFP-rDNA and pBR322-rDNA, we compared oxidability of pEGFP-rDNA plasmid and pEGFP vector (Figure 2(c)). The 8-oxodG content in DNA was analyzed using immunoassay on nitrocellulose membranes using antibodies to 8-oxodG conjugated with alkaline phosphatase. For measurement calibration, DNA samples with known 8-oxodG content were used (Figure 2(c), (1)). Plasmids pEGFP and pEGFP-rDNA were oxidized in 0.1% H_2_O_2_ solution at UV treatment (*λ* > 312 nm) or added for 1 h to the MCF7 culture medium. The plasmids were isolated from the cells as part of RNA fraction (see Methods) separating the high-molecular fraction of cellular DNA. Then hydrolysis was conducted using RNAse 1, and 8-oxodG content was assayed in DNA (Figure 2(c), (2)). In both experiments, pEGFP-rDNA plasmid contained more 8-oxodG than the pEGFP vector (Figure 2(c), (3)). After 1 h long incubation with cells, pEGFP-rDNA and pEGFP contained, respectively, 0.10 and 0.26 of 8-oxodG per one plasmid molecule. In other words, as a minimum, one 8-oxodG was contained in approximately one of four pEGFP-rDNA molecules and only one of 10 pEGFP molecules. One can suppose that rDNA contains still unidentified motifs, within which dG has even lower potential, than within the known GGG motif [21].

Thus, the compact single signals in the cellular cytoplasm (Figure 2(b)) and the considerable increase of 8-oxodG signal within the first hours of MCF7 incubation with GC-rDNA (Figure 2(a)) can be explained by stronger oxidation of GC-rDNA and, correspondingly, a higher rate of penetration of oxidized GC-rDNA fragments into the cells as compared to vectors.

Figure 6 shows the data on the localization of 8-oxodG in the cells exposed for 24 h to plasmids that carried and did not carry rDNA insertions. The localization of 8-oxodG in the presence of vectors pBR322 and pEGFP did not differ from the control cells: 8-oxodG signals were located in the cytoplasm (in mitochondrial DNA). However, in case of pEGFP-rDNA and pBR322-rDNA probes, we observed 8-oxodG in the nucleus (within the nucleolus area, Figure 6(c)) of approximately half of the cells.

Thus, oxidized GC-rDNA fragments penetrate the cells and are localized in the structures of nucleolus organizer regions. The appearance of extra rDNA fragments—potential competitors for binding to nucleolar proteins in the nucleolus—can result in a decline in the performance of the ribosome biogenesis.

### 3.6. GC-DNA Modulates the Performance of Ribosome Biogenesis in MCF7

For the quantification of intracellular 18S rRNA, the RT-qPCR technique was applied (Figure 6(d)). pEGFP-rDNA and pBR322-rDNA within the first hours after adding to the culture medium augmented the rRNA content in the cells by a factor of 1.5 to 2.0. pBR322 and pEGFP induced an increase of rRNA by 10%–15% (the difference from the controls is not significant, *p* > 0.05). After 72 h of cell exposure to pEGFP-rDNA and pBR322-rDNA, the rRNA content demonstrated a 2- or 3-fold decrease; pBR322 and pEGFP did not decrease or even increase the rRNA content in the cells.

### 3.7. The Expression of EGFP Gene from pEGFP and pEGFP-rDNA

**Figure 7:**
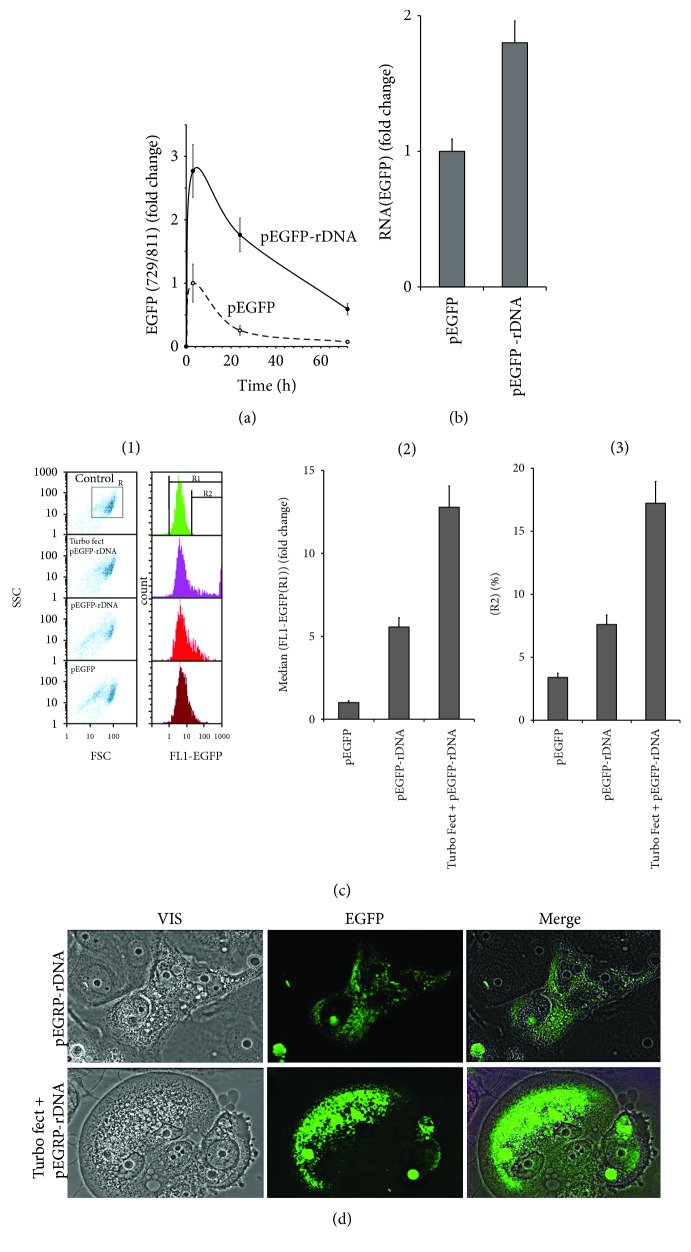
Expression of EGFP in MCF7 treated with pEGFP-rDNA or pEGFP for 24 h. (a) qPCR. Quantitation of pEGFP and pEGFP-rDNA in the cellular DNA after 3, 24, and 72 hours of incubation. pEGFP content (3 h) is taken as one unit. (b) RT-qPCR. The levels of EGFP-encoding RNAs in the cells exposed to pEGFP or pEGFP-rDNA. (c) FCA. (1)—nonfixed cell plots: SSC versus FCS; R—the analyzed cell subpopulation. Distribution of FL1-EGFP fluorescence intensities. R1- and R2-gated areas. (2)—median signal intensity of FL1-EGFP (gate R1). (3)—relative fractions of EGFP-positive cells (R2 gate). (d) FM-based evaluation of EGFP in the cells treated with pEGFP-rDNA or pEGFP-rDNA/Turbo Fect (×40).

The constructions pEGFP and pEGFP-rDNA contain CMV-promotor and *EGFP* gene (Figure 1), rendering a possibility to study EGFP expression in the cells. Successful expression requires DNA penetration into the cells. Therefore, we determined the rate of accumulation of pEGFP-rDNA and pEGFP plasmids inside the cells using qPCR (Figure 7(a)). Unlike the pEGFP vector, the pEGFP-rDNA accumulated inside the cells abundantly and could be detected even after 72 h.

After 24 h of cultivation in the presence of pEGFP-rDNA and pEGFP, the content of RNA *EGFP* increased (Figure 7(b)). The plasmid pEGFP-rDNA stimulated *EGFP* transcription in a greater degree, than pEGFP. We determined the amount of EGFP protein in the cells using flow cytometry (Figure 7(c)). For comparison, we performed pEGFP-rDNA plasmid transfection with the help of a Turbo Fect kit. We determined the ratio of highly fluorescent cells (gate R2, Figure 7(c), (1)) and mean fluorescence intensity of all the cells (gate R1). After 24 h, the cells cultivated in the presence of pEGFP-rDNA demonstrated a several times higher content of the EGFP protein, than in the presence of pEGFP vector, but lower, than in the case of transfection pEGFP-rDNA conducted with the aid of the Turbo Fect kit. In the presence of pEGFP-rDNA, the cell culture contained a larger fraction of cells with a relatively low amount of the EGFP protein. The data of fluorescence microscopy corroborated this conclusion (Figure 7(d)).

## 4. Discussion

### 4.1. GC-DNA Induces a Transient ROS Burst and Becomes Oxidized in MCF7

**Figure 8:**
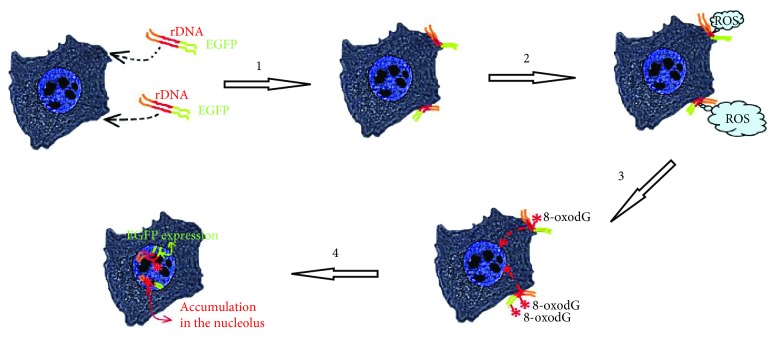
A summary of events that occur in MCF7 cells exposed to GC-DNA (pEGFP-rDNA as an example), which harbor genes that can be transcribed. (1)—GC-DNA approaches and interacts with the cell surface. (2)—GC-DNA stimulates a burst of ROS generation at the site of contact with the cell. ROS level is independent of the presence of easily oxidizable motifs. (3)—the easily oxidizable bases of rDNA (or other human easily oxidizable DNA sequences) are oxidized. GC-DNA without an easily oxidizable rDNA insert is oxidized to a lesser degree. (4)—oxidized GC-DNA is transfected into the cell nucleus and can be expressed (*EGFP* gene in pEGFP-rDNA molecules with intact promoter is expressed). GC-rDNA molecules are accumulated in the nucleolus.

The model GC-DNA, regardless of the presence of rDNA insertions, induced a ROS burst in the first hour of cultivation with the cells (Figure 4, scheme in Figure 8). The blood plasma cfDNA samples stimulate ROS synthesis as well; moreover, the higher rDNA content in the cfDNA is, the higher the ROS level is (Figure 4(a), (3)). Extremely intense ROS synthesis was observed in the places of the contact of GC-DNA fragments with the cells (Figure 4(b)). NADPH-oxidase NOX4, which plays an essential role in the cancer cell physiology [26, 27], is likely a key participant in this process. NOX4 catalyzes hydrogen peroxide synthesis in the cells. The NOX family enzymes are localized on cell membranes and in other places [28]. In the presence of GC-DNA, *NOX4* gene expression noticeably elevated at the levels of both transcription (Figure 5(b)) and translation (Figures 5(a) and 5(c)).

Both vectors stimulate NOX4 expression stronger and longer than plasmids with an rDNA insertion. This fact can be explained by the slower process of oxidation of the vector compared to rDNA (Figure 2). A negative feedback can be assumed that already oxidized cfDNA fragments send back to the cell a signal for reducing the ROS synthesis with NADPH-oxidase NOX4 enzyme. It can be hypothesized that the main objective (biological sense) of the elevation of ROS content in the presence of cfDNA is the oxidation of the latter in order to impart to cfDNA some novel biologic properties.

In the literature, the matter of influence of the DNA sequence on its oxidability is poorly explored. There are reports that within GGG motifs, deoxyguanosine has a very low oxidation potential, the minimum of all the bases [21]. In case of pEGFP-rDNA plasmid, an insertion of rDNA does not change significantly the ratio of GGG motif (Figure 1(b), (3)). However, the oxidation level of pEGFP-rDNA is notably higher, than that of pEGFP (Figure 2(c)). One can suppose that rDNA carries motifs with even lower oxidation potential. Besides, the type of oxidative modification may be also of importance. In recent years, great attention is paid to the oxidative modification of cytosine [29]. However, this is still unclear, if the oxidation potential of cytosine changed when cytosine is included in the GC-rich DNA regions and if the oxidation of cytosine has an effect on the oxidation potential of guanosine within the same GC-pair. This interesting issue needs further investigation.

### 4.2. GC-rDNA Has the Increased Property to Penetrate and Be Expressed in the Cancer Cells

CfDNA oxidation is a key event in the further development of cfDNA biological effects. The cfDNA fragments that harbour oxidized bases [18] penetrate inside the cell. The mechanism of penetration of oxidized DNA fragments through the cell membrane remains elusive. An existence of DNA sensors in the cells, which recognize the oxidized cfDNA bases, is suggested [18, 30, 31].

A number of facts suggest an elevated accumulation of GC-rDNA in MCF7 compared to the vectors. Sites of localization of GC-rDNA in the cells are more abundant, than sites of localization of the vector (Figure 3). The cells contain much more oxidized fragments of GC-rDNA, than oxidized fragments of the vectors (Figure 2(a)). GC-rDNA content in MCF7 is several times higher, than the vector content (Figure 7(a)). One of the major causes of more active transfection of MCF7 with GC-rDNA compared to the vectors is the increased oxidability of rDNA fragments (Figure 2(c)).

Nonoxidized DNA fragments have virtually no ability to pass through the cell membrane from the culture medium and be expressed in the cells. For efficient transfection of DNA, various techniques are usually applied. The exaggerated rate of GC-rDNA penetration into the cells also suggests a higher level of expression of genes carried by GC-rDNA. Indeed, we revealed a high level of expression of *EGFP* gene within pEGFP-rDNA even compared to the use of the common transfection technique (Figures 7(b), 7(c), and 7(d)) that correlated with the elevated content of pEGFP-rDNA in the cells (Figure 7(a)). Obviously, effective expression requires, in addition to DNA penetration to the cell, the intact promoter and gene itself. These conditions seem to be met in case of pEGFP-rDNA. The elevated oxidability of inserted rDNA guarantees rapid penetration of the plasmid into the cell; meanwhile, the promoter and gene itself become oxidized in a less number of plasmid molecules.

The above-mentioned possibility in principle to express genes from external cfDNA fragments is of importance for the understanding of processes of cancer cell survival during therapy. Earlier, we and other authors quoted data related to the influence of cfDNA on tumor therapy. CfDNA favors cancer cell survival under the conditions of deleterious impacts, evoking an adaptive response [18, 32].

### 4.3. Potential Use of the Study Findings in Antitumor Therapy

Thus, the total body of facts reported here suggests that GC-DNA could become a tool in antitumor therapy. Altering the properties of cfDNA could contribute to more effective tumor elimination and metastasis prevention. Several approaches can be suggested.

#### 4.3.1. Modulation of the Transcriptional Activity of Tumor Genes

Data shown in Figure 6 suggest a possibility of penetration of oxidized cf-rDNA fragments into the cell nucleus. When they reached the nucleus, rDNA fragments interact with the nucleolar proteins, which possess binding selectivity with respect to rDNA motifs. Moreover, cf-rDNA fragments can compete with the nuclear ribosomal genes for binding to and exhaust the cell pool of factors necessary for the ribosome biogenesis. An indirect evidence in favor of this speculation is the decline of rRNA content in the cells cultivated in the presence of GC-rDNA, but not plasmid vectors (Figure 6(b)). Other motifs within GC-cfDNA could be expected to modulate expression of other genes by competing for binding to transcription factors in the respective nuclear areas.

Transfection effectiveness of plasmids carrying easily oxidizable regions and motifs, which can affect the expression of the tumor genes, can be increased via preliminary oxidation of the plasmids *in vitro*. The modern methods allow selective oxidation of DNA bases with different oxidation potentials [33]. Such conditions can be found, when easily oxidizable DNA fragments only are oxidized.

#### 4.3.2. Blocking Adaptive cfDNA Action on Tumor Cells

Earlier cfDNA was shown to induce an adaptive response, which increases the survival rate of cancer cells, during chemotherapy [18, 32]. Oxidized DNA induces much higher adaptive response than unoxidized. The adaptive response is induced by a short-time elevation of ROS synthesis under the action of cfDNA [32]. The elevated ROS level induces a network of DNA damage response (DDR) mechanisms that counteract toxicity and safeguard genome integrity [34]. If the ROS burst is blocked, then the adaptive response does not develop [18]. In this context, the use of natural low-toxic antioxidants, which quench ROS and have anticancer properties, seems a perspective [35].

CfDNA can carry mutant genes from the tumor genome, which trigger cancerogenesis in healthy cells. Penetration of cfDNA into the healthy cells can result in metastases. Previously, we showed that GC-DNA considerably altered the functional activity of human stem cells [15]. Antioxidants, which prevent cfDNA oxidation, can thus preclude its penetration to the healthy cells.

## Data Availability

The data used to support the findings of this study are available from the corresponding author upon request.

## Abbreviations

CfDNA: Cell-free DNA of MCF7 culture medium
GC-cfDNA: GC-rich fragments of cell-free DNA
GC-DNA: A generic term for all DNA probes used in this study
GC-rDNA: Plasmids carrying rDNA insertions.

## References

[1] Aucamp J., Bronkhorst A. J., Badenhorst C. P. S., Pretorius P. J. (2016). A historical and evolutionary perspective on the biological significance of circulating DNA and extracellular vesicles. *Cellular and Molecular Life Sciences*.

[2] Suzuki N., Kamataki A., Yamaki J., Homma Y. (2008). Characterization of circulating DNA in healthy human plasma. *Clinica Chimica Acta*.

[3] Lander E. S., Linton L. M., Birren B. (2001). Initial sequencing and analysis of the human genome. *Nature*.

[4] Krapf F. E., Herrmann M., Leitmann W., Kalden J. R. (1989). Are retroviruses involved in the pathogenesis of SLE? Evidence demonstrated by molecular analysis of nucleic acids from SLE patients’ plasma. *Rheumatology International*.

[5] Alvarado-Vásquez N. (2015). Circulating cell-free mitochondrial DNA as the probable inducer of early endothelial dysfunction in the prediabetic patient. *Experimental Gerontology*.

[6] Tuboly E., Mcllroy D., Briggs G., Lott N., Balogh Z. J. (2017). Clinical implications and pathological associations of circulating mitochondrial DNA. *Frontiers in Bioscience*.

[7] Veiko N. N., Bulycheva N. A., Roginko O. A. (2008). Ribosomal repeat in cell free DNA as a marker for cell death. *Biochemistry*.

[8] Veiko N. N., Shubaeva N. O., Ivanova S. M., Speranskii A. I., Lyapunova N. A., Spitkovskii D. M. (2006). Blood serum DNA in patients with rheumatoid arthritis is considerably enriched with fragments of ribosomal repeats containing immunostimulatory CpG-motifs. *Bulletin of Experimental Biology and Medicine*.

[9] Veiko N. N., Konorova I. L., Neverova M. E. (2010). The effect of CpG-rich DNA fragments on the development of hypertension in spontaneously hypertensive rats (SHR). *Biochemistry*.

[10] Korzeneva I. B., Kostuyk S. V., Ershova E. S. (2016). Human circulating ribosomal DNA content significantly increases while circulating satellite III (1q12) content decreases under chronic occupational exposure to low-dose gamma- neutron and tritium beta-radiation. *Mutation Research/Fundamental and Molecular Mechanisms of Mutagenesis*.

[11] Chestkov I. V., Veiko N. N., Ershova L. S. (2016). The method for analysis of the copy number variations of GC-rich repeat of human genome in damaged DNA. Detection of increasing copy number of ribosomal genes in extracellular DNA circulating in blood plasma of smokers’ humans. *Medical Genetics*.

[12] Kostyuk S. V., Alekseeva A. Y., Kon’kova M. S. (2014). Oxidized extracellular DNA suppresses nitric oxide production by endothelial NO synthase (eNOS) in human endothelial cells (HUVEC). *Bulletin of Experimental Biology and Medicine*.

[13] Bulicheva N., Fidelina O., Mkrtumova N. (2008). Effect of cell-free DNA of patients with cardiomyopathy and rDNA on the frequency of contraction of electrically paced neonatal rat ventricular myocytes in culture. *Annals of the New York Academy of Sciences*.

[14] Efremova L. V., Kostyuk S. V., Khaspekov L. G., Veiko N. N., Gahan P. B. (2010). Accumulating fragments of extracellular DNA (ecDNA) influence rat primary cerebellum granule cell culture. *Circulating Nucleic Acids in Plasma and Serum*.

[15] Kostyuk S., Smirnova T., Kameneva L. (2015). GC-rich extracellular DNA induces oxidative stress, double-strand DNA breaks, and DNA damage response in human adipose-derived mesenchymal stem cells. *Oxidative Medicine and Cellular Longevity*.

[16] Speranskii A. I., Kostyuk S. V., Kalashnikova E. A., Veiko N. N. (2015). Erichment of extracellular DNA from the cultivation medium of human peripheral blood mononuclears with genomic CpG rich fragments results in increased cell production of IL6 and TNF*α* via activation of the NF-*κ*B signaling pathway. *Biochemistry*.

[17] Aarthy R., Mani S., Velusami S., Sundarsingh S., Rajkumar T. (2015). Role of circulating cell-free DNA in cancers. *Molecular Diagnosis & Therapy*.

[18] Kostyuk S. V., Konkova M. S., Ershova E. S. (2013). An exposure to the oxidized DNA enhances both instability of genome and survival in cancer cells. *PLoS One*.

[19] Ma R., Zhao J., Du H. C., Tian S., Li L. W. (2012). Removing endotoxin from plasmid samples by Triton X-114 isothermal extraction. *Analytical Biochemistry*.

[20] Ershova E. S., Jestkova E. M., Chestkov I. V. (2017). Quantification of cell-free DNA in blood plasma and DNA damage degree in lymphocytes to evaluate dysregulation of apoptosis in schizophrenia patients. *Journal of Psychiatric Research*.

[21] von Sonntag C. (2006). *Free-Radical-Induced DNA Damage and Its Repair. A Chemical Perspective*.

[22] Kostyuk S. V., Kvasha M. A., Khrabrova D. A. (2018). Symmetric dimeric bisbenzimidazoles DBP(n) reduce methylation of *RARB* and *PTEN* while significantly increase methylation of rRNA genes in MCF-7 cancer cells. *PLoS One*.

[23] LeBel C. P., Ischiropoulos H., Bondy S. C. (1992). Evaluation of the probe 2',7'-dichlorofluorescin as an indicator of reactive oxygen species formation and oxidative stress. *Chemical Research in Toxicology*.

[24] Rykova E. Y., Morozkin E. S., Ponomaryova A. A. (2012). Cell-free and cell-bound circulating nucleic acid complexes: mechanisms of generation, concentration and content. *Expert Opinion on Biological Therapy*.

[25] Bryzgunova O. E., Tamkovich S. N., Cherepanova A. V. (2015). Redistribution of free- and cell-surface-bound DNA in blood of benign and malignant prostate tumor patients. *Acta Naturae*.

[26] Shanmugasundaram K., Nayak B. K., Friedrichs W. E., Kaushik D., Rodriguez R., Block K. (2017). NOX4 functions as a mitochondrial energetic sensor coupling cancer metabolic reprogramming to drug resistance. *Nature Communications*.

[27] Tobar N., Guerrero J., Smith P. C., Martínez J. (2010). NOX4-dependent ROS production by stromal mammary cells modulates epithelial MCF-7 cell migration. *British Journal of Cancer*.

[28] Zhang L., Nguyen M. V. C., Lardy B. (2011). New insight into the Nox4 subcellular localization in HEK293 cells: first monoclonal antibodies against Nox4. *Biochimie*.

[29] Äijö T., Bonneau R., Lähdesmäki H. (2018). Generative models for quantification of DNA modifications. *Methods in Molecular Biology*.

[30] Ermakov A. V., Konkova M. S., Kostyuk S. V., Izevskaya V. L., Baranova A., Veiko N. N. (2013). Oxidized extracellular DNA as a stress signal in human cells. *Oxidative Medicine and Cellular Longevity*.

[31] Glebova K., Veiko N., Kostyuk S., Izhevskaya V., Baranova A. (2015). Oxidized extracellular DNA as a stress signal that may modify response to anticancer therapy. *Cancer Letters*.

[32] Anunobi R., Boone B. A., Cheh N. (2018). Extracellular DNA promotes colorectal tumor cell survival after cytotoxic chemotherapy. *The Journal of Surgical Research*.

[33] Chang Z., Yang Y., He J., Rusling J. F. (2018). Gold nanocatalysts supported on carbon for electrocatalytic oxidation of organic molecules including guanines in DNA. *Dalton Transactions*.

[34] Mullenders L. H. F. (2018). Solar UV damage to cellular DNA: from mechanisms to biological effects. *Photochemical & Photobiological Sciences*.

[35] Mahgoub A. M., Mahmoud M. G., Selim M. S., EL Awady M. E. (2018). Exopolysaccharide from marine Bacillus velezensis MHM3 induces apoptosis of human breast cancer MCF-7 cells through a mitochondrial pathway. *Asian Pacific Journal of Cancer Prevention*.

